# Multitarget Acaricidal Activity of Essential Oils from *Alpinia zerumbet* and *Mesosphaerum suaveolens* Against *Tetranychus urticae*

**DOI:** 10.3390/insects16111119

**Published:** 2025-11-02

**Authors:** Herus Pablo Firmino Martins, Giovana Lopes da Silva, Erika Pereira da Silva, Mariano Oscar Anibal Ibanez Rojas, Francisco José Teixeira Gonçalves, Izaias Santos Marques, Marcos Vinícius de Sousa Negreiros, Victório Alessandro de Leão Loeschke Morais, Franciele Costa de Sousa, Antonio José Cantanhede Filho, Bruno de Araújo Gomes, Edson Rodrigues Filho, Solange Maria de França, Adalberto Hipólito de Sousa, Lucas Martins Lopes, Gutierres Nelson Silva, Douglas Rafael e Silva Barbosa

**Affiliations:** 1 Instituto Federal do Maranhão, Campus Codó, Codó 65400-000, MA, Brazil; herus.pablo@acad.ifma.edu.br (H.P.F.M.); giovana.silva@ifma.edu.br (G.L.d.S.); erika.silva@acad.ifma.edu.br (E.P.d.S.); ibanez@ifma.edu.br (M.O.A.I.R.); jose.teixeira@ifma.edu.br (F.J.T.G.); izaias-marques@hotmail.com (I.S.M.); marcosn@acad.ifma.edu.br (M.V.d.S.N.); victorio.m@acad.ifma.edu.br (V.A.d.L.L.M.); 2 Programa de Pós-Graduação em Agronomia, Universidade Federal do Piauí, Teresina 64049-550, PI, Brazil; souzahfrancy@gmail.com; 3 Instituto Federal do Maranhão, Campus Monte Castelo, São Luís 65030-005, MA, Brazil; prof.antoniofilho@ifma.edu.br (A.J.C.F.); bruno.gomes@ifma.edu.br (B.d.A.G.); 4 Departamento de Química, Universidade Federal de São Carlos, São Carlos 13565-905, SP, Brazil; edinho.labiommi@gmail.com; 5 Departamento de Agronomia, Universidade Estadual do Maranhão, Balsas 65800-000, MA, Brazil; solangefranca@professor.uema.br; 6 Centro de Ciências Biológicas e da Natureza, Universidade Federal do Acre, Rio Branco 69920-900, AC, Brazil; adalberto.sousa@ufac.br; 7 Instituto Federal do Amazonas, Campus Eirunepé, Eirunepé 69020-120, AM, Brazil; lucas.lopes@ifam.edu.br; 8 Instituto Federal do Mato Grosso do Sul, Campus Nova Andradina, Nova Andradina 79750-000, MS, Brazil; gutierres.silva@ifms.edu.br

**Keywords:** bioacaricide, integrated control, two-spotted spider mite, sublethal effects, toxicity

## Abstract

The two-spotted spider mite, *Tetranychus urticae*, is a global pest with increasing resistance to conventional acaricides, prompting the search for sustainable alternatives. Essential oils (EOs) are promising botanical biocides due to ecological safety and multitarget action. We evaluated lethal and sublethal effects of EOs from *Alpinia zerumbet* and *Mesosphaerum suaveolens* against *T. urticae*. Oils were obtained by hydrodistillation and characterized by GC–MS (major constituents: *A. zerumbet*—1,8-cineole 14.05%, sabinene 12.6%; *M. suaveolens*—β-sabinene (predominant), spathulenol 12.28%, 1,8-cineole 11.01%). In adult bioassays, *M. suaveolens* was more toxic (LC_50_ = 4.24 µL mL^−1^), whereas *A. zerumbet* showed LC_50_ = 8.74 and LC_90_ = 46.24 µL mL^−1^. In ovicidal assays at LC_90_, egg viability declined to 2% with *A. zerumbet* versus 57% with *M. suaveolens*. Repellency at sublethal concentrations (LC_20_–LC_30_) was high for both oils (≥75%) and exceeded 90% for *M. suaveolens*. Both oils suppressed population growth (instantaneous rate *r_i_* reduced from 0.5848 in the control to 0.4746–0.5155 under treatments). PCA confirmed lethal concentration and repellency as the main discriminators among treatments. These data demonstrate the multitarget potential of *A. zerumbet* and *M. suaveolens* EOs as botanical acaricides for sustainable management of *T. urticae*.

## Simple Summary

The two-spotted spider mite (*Tetranychus urticae*) harms many crops and rapidly develops resistance to pesticides. To evaluate safer options, we tested essential oils from *Alpinia zerumbet* (“jardineira”) and *Mesosphaerum suaveolens* (“bamburral”), common in Brazil. We characterized their main constituents and assessed activity in the laboratory. Both oils killed adults, reduced egg hatch, repelled mites at sublethal doses, and slowed population growth. *M. suaveolens* oil was more potent against adults, whereas *A. zerumbet* was stronger against eggs. These findings support these oils as promising, sustainable tools for mite management, potentially reducing reliance on synthetic acaricides.

## 1. Introduction

The two-spotted spider mite (*Tetranychus urticae* Koch) is a cosmopolitan, highly polyphagous species that infests a wide range of agricultural and ornamental plants, with more than 1100 documented hosts worldwide [1]. It causes substantial yield losses through feeding that damages plant tissues, reduces photosynthetic activity, and promotes premature leaf abscission. Its high reproductive capacity, short lifespan, and arrhenotokous reproduction also enable rapid population increases in agroecosystems [2]. Although chemical control remains the primary approach for managing *T. urticae*, field efficacy is increasingly undermined by rapid, multi-acaricide resistance and by side effects that can trigger resurgence [3,4], reinforcing the need for sustainable alternatives.

Botanical pesticides have gained attention for their biocidal activity and low environmental persistence, offering a viable option within integrated pest management (IPM) programs. Essential oils (EOs), particularly rich in mono- and sesquiterpenes, can confer toxic, repellent, and ovicidal properties and may reduce non-target impacts when appropriately formulated and dosed [5]. For *T. urticae* specifically, several EOs or EO-based formulations have shown adulticidal effects, reductions in egg hatch, and adverse impacts on population parameters, supporting their potential as acaricidal tools [6,7,8]. However, important gaps remain: most reports emphasize acute adult mortality; integrated assessments quantifying ovicidal activity, repellency at sublethal doses, and consequences for population growth are less common; and few studies explicitly link GC–MS chemical profiles to bioactivity to guide formulation.

*Alpinia zerumbet* (Zingiberaceae; “jardineira”) is traditionally used for medicinal and aromatic purposes, and its leaf oil is commonly characterized by constituents such as 1,8-cineole and sabinene, among others [9]. *Mesosphaerum suaveolens* (Lamiaceae; “bamburral”) is widely used in Brazilian folk medicine, and its oil can include β-sabinene, 1,8-cineole, and oxygenated sesquiterpenes [10]. Although EO bioactivity against *T. urticae* has been documented in general, to our knowledge there are no prior reports evaluating the acaricidal efficacy of *A. zerumbet* or *M. suaveolens* essential oils against *T. urticae*. This gap limits our understanding of whether the chemotypes of these widely available plants can be leveraged for mite management.

Accordingly, this study combined GC–MS profiling with bioassays to quantify (i) adult female toxicity (LC_50_/LC_90_), (ii) ovicidal effects, (iii) repellency at sublethal concentrations (LC_20_/LC_30_), and (iv) impacts on population growth. This integrated evaluation provides the first evidence for the activity of these two plant oils against *T. urticae* and clarifies their comparative strengths across life stages and response endpoints, informing subsequent formulation and IPM deployment.

## 2. Materials and Methods

Experiments were conducted at the Multidisciplinary Laboratory of the Instituto Federal de Educação, Ciência e Tecnologia do Maranhão (IFMA), Campus Codó, in a climatic chamber maintained at 25 ± 1 °C, 70 ± 10% relative humidity, and a 12 h photoperiod. Chromatographic analyses of the essential oils were performed at the Laboratory of Microorganism Micromolecular Biochemistry, Federal University of São Carlos (UFSCar).

### 2.1. Collection of Plants for Essential Oil Extraction

Plant material for essential-oil extraction comprised *A. zerumbet* (“jardineira”) and *M. suaveolens* (“bamburral”), both recognized for chemical properties with potential acaricidal activity. Collections were carried out in the municipality of Codó, Maranhão, Brazil (4.45562° S, 43.89240° W), approximately 217 km (straight-line distance) from the state capital, São Luís.

### 2.2. Essential Oil Extraction

Essential oils were obtained from fully expanded, healthy leaves of *Alpinia zerumbet* and from leaves plus tender leafy stems of *Mesosphaerum suaveolens* (used because the leaves are very small) by hydrodistillation using a modified Clevenger-type apparatus fitted with a condenser. Only fresh, undamaged, disease-free material was used; field debris was gently removed (brief rinse and blot dry), and tissues were coarsely chopped immediately before loading. Plant material was processed on the day of collection, avoiding senescent or wilted tissues. The plant material was coarsely ground, loaded into the apparatus, and distilled for 3 h at gentle reflux. The recovered oil was then collected and dried over analytical-grade anhydrous sodium sulfate (Na_2_SO_4_) to remove residual moisture. After drying, oils were transferred to amber glass vials with PTFE-lined caps and stored at 4 °C, protected from light, until GC–MS analysis and bioassays.

### 2.3. Chemical Profiling of Essential Oils by GC-MS

The chemical composition of the essential oils was determined by gas chromatography–mass spectrometry (GC–MS). Analyses were performed on a Shimadzu QP2010 Plus (Shimadzu Corporation, Kyoto, Japan) (scan mode, *m*/*z* 40–650) equipped with an Rxi-5ms capillary column (10 m × 0.10 mm × 0.10 μm). The injector operated in splitless mode at 250 °C. The oven program started at 40 °C (1 min hold), increased at 35 °C min^−1^ to 320 °C, and was held at 320 °C for 1 min. Helium was used as the carrier gas at a constant flow of 0.45 mL min^−1^.

The mass spectrometer operated with 70 eV electron ionization, an interface temperature of 250 °C, and an ion source temperature of 200 °C. Quantitative data were obtained as relative peak-area percentages using the area-normalization method.

To ensure optimal system performance and accurate assignment of linear retention indices (LRI) for each compound, a standard mixture of n-alkanes (C7–C50; Sigma-Aldrich, St. Louis, MO, USA)) was analyzed under the same chromatographic conditions. A 1 μL aliquot of this standard was injected, and the corresponding retention times, together with those of the target compounds, were used as external references for LRI calculation.

LRI values were automatically calculated using GCMSolution, obviating manual computation with the Van den Dool and Kratz equation. These values were then compared with literature data obtained on columns of similar polarity to the Rxi-5ms stationary phase.

Compound identification was based on comparison of the acquired mass spectra with reference spectra in the NIST database. Literature LRI values were also consulted to confirm identifications. A tolerance of ±100 LRI units was considered acceptable, corresponding to the retention difference expected for one additional carbon atom in the homologous series.

### 2.4. Rearing of T. urticae

The *T. urticae* colony was established on jack bean (*Canavalia ensiformis* L.) grown in 5 L pots containing a 3:1 soil–humus mixture. The source population of *T. urticae* was collected in February 2020 from cowpea (*Vigna unguiculata* (L.) Walp.) in Teresina, Piauí, Brazil (latitude—5.08917, longitude—42.80194). For colony maintenance, 25-day-old plants were infested with eggs, larvae, nymphs, and adults of the two-spotted spider mite. The stock colony was not exposed to essential oils and was maintained under controlled conditions (25 ± 1 °C, 70 ± 10% relative humidity, 12 h photoperiod) [11], and was propagated in the laboratory and greenhouse for several generations prior to bioassays.

### 2.5. Lethal Effect of Essential Oils on Adult Females of T. urticae

The toxicity of the essential oils to *T. urticae* was evaluated using a residual contact bioassay. Jack bean leaf disks (5.0 cm diameter) were immersed, under gentle agitation for 30 s, in solutions of essential oil or in the control solution (distilled water with 2% dimethyl sulfoxide, DMSO). The concentrations for *M. suaveolens* were 0.8, 1.6, 6.4, 19.2, and 26.0 µL mL^−1^, determined from preliminary range-finding tests; for *A. zerumbet*: 3.2, 6.4, 12.8, 19.2, and 26.0 µL mL^−1^. After air-drying for 30 min, each disk was infested with 10 adult females (4–5 days old) of *T. urticae* [11].

The bioassay followed a completely randomized design with five leaf disks per concentration (each disk = one Petri dish, 90 × 15 mm; 10 adult females/disk; total 50 mites per concentration per run). The entire experiment was conducted in three independent runs on different dates, each run comprising the same five leaf disks per concentration to account for temporal and dilution variability.

Leaf disks were placed with the abaxial surface facing upward on filter paper positioned over a water-saturated sponge inside plastic Petri dishes. A central hole in each lid was covered with voile fabric to allow gas exchange. The dishes were maintained in a climatic chamber at 25 ± 1 °C, 70 ± 10% relative humidity, and a 12 h photoperiod. Mortality was assessed 48 h after infestation; mites were considered dead if they failed to move when gently probed with a fine-bristle brush.

### 2.6. Lethal Effect of Essential Oils on T. urticae Eggs

Leaf disks of jack bean (*C. ensiformis*) measuring 5.0 cm in diameter were infested with 5 adult females of *T. urticae* (4–5 days old), which were allowed to oviposit for 48 h. After this period, 20 eggs were selected per disk and immersed in the LC_50_ and LC_90_ concentrations of each essential oil (previously determined in the residual contact bioassay), along with a control treatment consisting of distilled water + DMSO. The procedure followed the same methodology used for evaluating the lethal effect on adult females. Ten replicates were performed for each lethal concentration and for the control. Egg viability was assessed daily by counting the number of hatched larvae until hatching was complete.

### 2.7. Instantaneous Population Growth Rate

The instantaneous population growth rate (ri) for the LC_30_ and LC_20_ concentrations (previously determined in the residual contact bioassay) was calculated for adult females, following the same methodology used in the residual effect test. A total of 10 replicates were performed for each treatment, including a control group treated with distilled water + DMSO. The calculation was based on the equation [12]:

ri = [ln(Nf/N_0_)]/Δt

where

Nf = Final number of mites

N_0_ = Initial number of mites

Δt = Number of days over which the assay was conducted

The total evaluation period was 10 days after the beginning of the experiment, during which all developmental stages were counted (eggs, mobile immature stages, and adults).

### 2.8. Repellent Effect of Essential Oils

Repellency tests were conducted using arenas made of plastic Petri dishes (90 × 15 mm) filled with a 1 cm thick moistened sponge encased in filter paper. The sublethal concentrations utilized (LC_30_ and LC_20_) were previously determined in toxicity evaluations of adult females.

Jack bean leaf disks (5.0 cm in diameter) were partially immersed, with one half dipped in the essential oil solution (essential oil + DMSO) and the opposite half in distilled water + DMSO (control). The central portion of the disk (main midrib) was not submerged in either solution and thus remained untreated. Mites were released directly along the midrib, ensuring that individuals initially occupied a neutral area free from any contact with the test substances. This configuration allowed for equal opportunity for the mites to move toward either the treated or untreated side. The solution preparation and infestation followed the same protocols as the toxicity test, which entailed the introduction of 10 mature female *T. urticae* (4–5 days old) into the center of each disk. The number of females present in each treatment zone was assessed 48 h after infestation.

To assess the repellent effect, the repellency index (RI) was calculated using the formula: RI = 2G/(G + P), where G signifies the number of mites attracted to the treatment and P indicates the number of mites attracted to the control. The criterion for evaluating the treatment’s repellent efficacy was determined by the mean repellency index (RI) and its standard deviation (SD). If the mean RI is less than 1 − SD, the oil is classified as repellent; if it is greater than 1 + SD, the oil is classified as attracting; and if it falls between 1 − SD and 1 + SD, the oil is classified as neutral [13]. Each treatment was subjected to testing with ten replicates.

### 2.9. Multivariate Assessment of Lethal and Sublethal Effects on T. urticae: PCA of Experimental Parameters

A Principal Component Analysis (PCA) was conducted to ascertain the relative contributions of the examined parameters—lethal concentration, egg viability, repellency, and population growth rate—to the overall management of *T. urticae*. This multivariate method was employed to consolidate the biological effects assessed in the study and to substantiate the ultimate interpretation of treatment efficacy.

For PCA, we assembled a treatment × variable matrix using one summary metric per endpoint to avoid redundancy and collinearity: LC_50_ (adult toxicity), egg hatchability (% viable), repellency index (two-choice test), and instantaneous population growth rate. To ensure that higher values consistently reflected greater efficacy, we reversed the direction of LC metrics by multiplying LC_50_ by −1 (i.e., lower LC → higher efficacy). All variables were centered and scaled to unit variance (z-scores) prior to analysis.

We verified the suitability of PCA by inspecting the correlation structure among variables and conducting Bartlett’s test of sphericity (*p* < 0.05 as criterion). The analysis was run in R (base prcomp), with components retained based on the scree plot and Kaiser’s criterion (eigenvalues > 1). We report the proportion of variance explained, loadings, and scores, and interpret loadings to identify which endpoints most strongly drive treatment separation.

The PCA facilitated a visual and statistical evaluation of the impact of various treatments on the biological response of *T. urticae*, as well as the identification of the variables most significantly correlated with treatment differentiation. This multivariate integration established a solid foundation for comprehending the combined fatal and sublethal effects of the essential oils, augmenting the univariate analyses and reinforcing the study’s conclusions.

### 2.10. Statistical Analyses

All experiments were conducted under a completely randomized design, with the replicate structure described in the corresponding bioassay subsections.

Probit analyses. The lethal (LC_50_ and LC_90_) and sublethal (LC_30_ and LC_20_) concentrations of the essential oils were estimated by probit regression using the PROC PROBIT procedure in SAS software version 8.02 [14]. Models used binomial responses (number dead, number exposed) and a probit link, with log_10_-transformed concentration as the predictor. Control mortality was ≤5% across assays; therefore, no Abbott correction was required. We report slope ± SE, χ^2^ goodness-of-fit, and LC estimates with 95% fiducial limits. Overdispersion was evaluated via Pearson χ^2^/df; when heterogeneity was detected, the heterogeneity factor was used to adjust confidence limits. Parallelism of dose–response slopes between oils was examined; when slopes did not differ (*p* > 0.05), we compared potencies directly; otherwise, LC estimates were interpreted within oil. Toxicity Ratios (TR) were calculated by dividing the LC_50_ and/or LC_90_ of the less toxic oil by the corresponding value of the more toxic oil; differences were considered meaningful when 95% fiducial limits of LC estimates did not overlap (and TR ≈ 1 was not supported).

Ovicidal effect and population growth. For the ovicidal effect (egg hatchability) and instantaneous population growth rate, data were subjected to analysis of variance (ANOVA), and means were compared using Tukey’s test at a 5% significance level. Assumptions were checked on model residuals (Shapiro–Wilk for normality; Levene’s test for homoscedasticity) and were satisfied; accordingly, no data transformation was applied. Results are presented as means ± SE.

Repellency. Repellency was assessed by comparing the number of mites attracted to each treatment using the Chi-square test, implemented through PROC FREQ in SAS. Expected cell frequencies met χ^2^-approximation requirements; residual/fit diagnostics were inspected to confirm test validity.

Multivariate integration. Additionally, a PCA was performed in R to integrate the different biological parameters evaluated—using the variable set and preprocessing described in Section 2.9—to ascertain the variables that most significantly contributed to the distinction among treatments and to enhance the overall interpretation of the data concerning mite control. Unless stated otherwise, statistical significance was set at α = 0.05, and all *p*-values are two-tailed.

## 3. Results

### 3.1. Chemical Composition of Essential Oils

The chemical analysis of *A. zerumbet* essential oil via gas chromatography–mass spectrometry (GC–MS) demonstrated a preponderance of terpenoid components. The primary components were 1,8-cineol (14.05%) and sabinene (12.6%), succeeded by β-eudesmol (4.5%), β-cymene (6.87%), and caryophyllene oxide (2.71%) (Table 1). The results demonstrate that the oil predominantly consists of monoterpenes—both oxygenated (e.g., 1,8-cineol) and hydrocarbon variants (e.g., sabinene)—as well as oxygenated sesquiterpenes, indicated by the detection of β-eudesmol and caryophyllene oxide.

The essential oil of *M. suaveolens* exhibited a varied composition of terpenoid chemicals. The predominant component was beta-sabinene (15.78%), succeeded by spathulenol (12.28%), 1,8-cineole (11.01%), β-bourbonene (11.1%), and β-eudesmol (8.52%) (Table 2). The results reveal a chemical composition predominantly characterized by monoterpenes, especially beta-sabinene and 1,8-cineole, with oxygenated sesquiterpenes such spathulenol and β-eudesmol. Other compounds, such as elemol, viridiflorol, and fenchol, were also identified in lower concentrations, further reinforcing the presence of sesquiterpenes in the oil.

### 3.2. Lethal Effect of Essential Oils on Adult Females of T. urticae

In the toxicity assessment of essential oils, the oil extracted from *M. suaveolens* exhibited the lowest lethal concentration (LC_50_) value, estimated at 4.24 µL/mL, with a 95% confidence interval (CI_95_%) ranging from 3.41 to 5.25 µL/mL. The LC_90_ for this oil was 15.08 µL/mL (CI_95_%: 11.50–21.49 µL/mL) (Table 3). In contrast, the essential oil of *A. zerumbet* showed higher LC_50_ and LC_90_ values of 8.74 µL/mL and 46.24 µL/mL, respectively (Table 3).

Sublethal concentrations (LC_20_ and LC_30_) were also determined and used to assess the sublethal effects of the oils. For *A. zerumbet*, the LC_20_ and LC_30_ were 2.92 and 4.42 µL/mL, respectively, while for *M. suaveolens*, they were 1.84 and 2.52 µL/mL.

**Figure 1:**
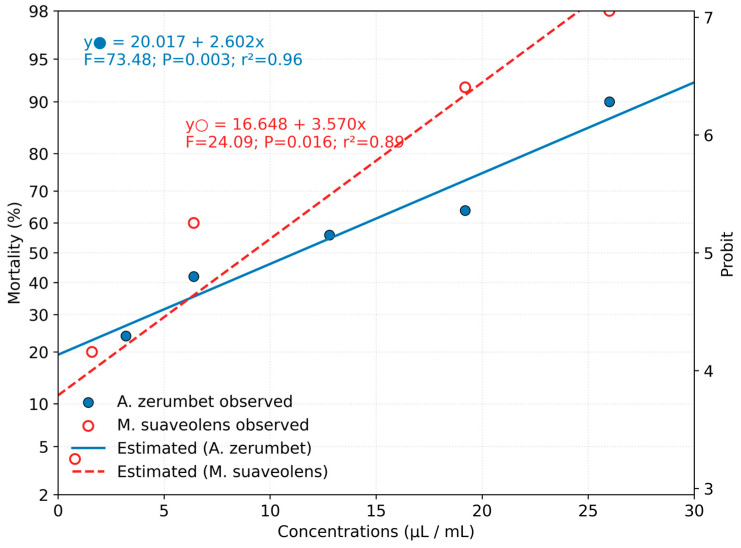
Dose–response curves for the essential oils of *A. zerumbet* and *M. suaveolens* on *T. urticae* females based on Probit analysis.

A comparative analysis of toxicity between the oils revealed significant differences, as evidenced by the non-overlapping confidence intervals of their lethal concentrations, indicating that *M. suaveolens* oil was significantly more toxic than that of *A. zerumbet*. The Probit model provided a good fit for the concentration–mortality data, with *p*-values greater than 0.05, confirming the adequacy of the regression model. The dose–response relationship was well represented by the regression line, which can be used to estimate the required oil concentration to increase mortality rates (Figure 1). According to the regression equation, for *A. zerumbet* oil, an increase of 1 µL/mL in concentration leads to a 2.60% increase in mite mortality, while for *M. suaveolens*, this increase is estimated at 3.57%.

### 3.3. Lethal Effect of Essential Oils on T. urticae Eggs

**Figure 2:**
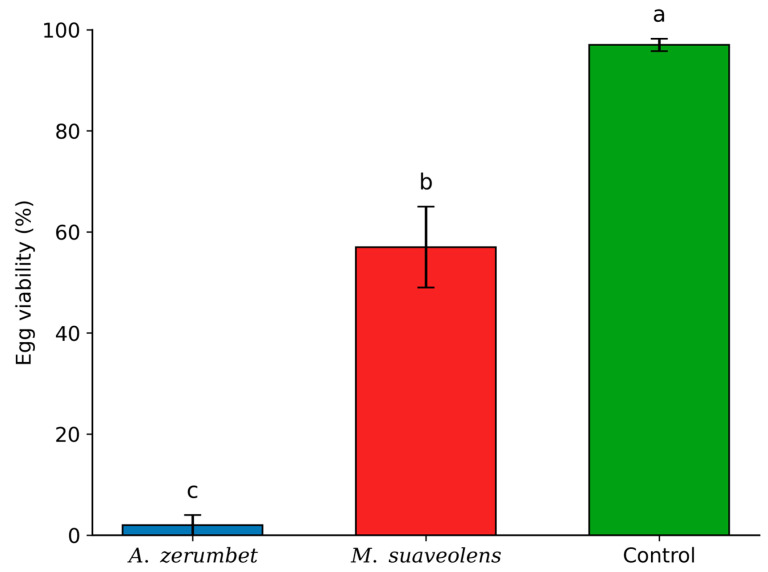
Egg viability (%) of *T. urticae* after exposure to the LC_90_ concentrations of *A. zerumbet* and *M. suaveolens* essential oils. Bars followed by different letters differ significantly according to Tukey’s test at 5% probability. n = 400; *p* < 0.0001.

The evaluation of the lethal effects of the essential oils of *A. zerumbet* and *M.suaveolens* on *Tetranychus urticae* eggs revealed that both oils significantly reduced egg hatchability. Although significant differences were observed between the tested concentrations and the control, these differences were evident only at the LC_90_ level (Figure 2). At the LC_50_ concentration, there was no significant difference among the effects of *A. zerumbet*, *M. suaveolens*, and the untreated control.

At LC_90_, *A. zerumbet* essential oil showed the most pronounced ovicidal effect, reducing egg viability to only 2%. In contrast, *M. suaveolens* oil resulted in 57% egg viability (Figure 2), indicating a superior ovicidal efficacy of *A. zerumbet* essential oil under the tested conditions.

### 3.4. Instantaneous Population Growth Rate

**Figure 3:**
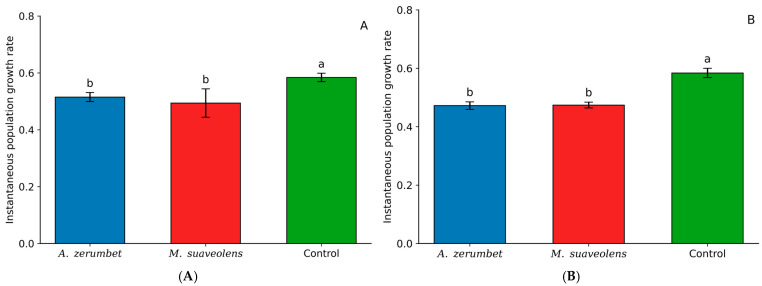
Instantaneous population growth rate (ri) of *T. urticae* after exposure to LC_20_ (**A**) and LC_30_ (**B**) concentrations of *A. zerumbet* and *M. suaveolens* essential oils. Bars followed by different letters differ significantly according to Tukey’s test at 5% probability. n = 200; *p* < 0.0001.

When evaluating the effects of LC_20_ concentrations of *A. zerumbet* (0.5155) and *M. suaveolens* (0.4984) essential oils, no significant difference was observed between the two treatments. However, both resulted in a reduced population growth rate compared to the control treatment (0.5848) (Figure 3A). A similar trend was observed at the LC_30_ concentration, with comparable results; nonetheless, *M. suaveolens* (0.4746) showed a slightly lower population growth rate than that observed at its LC_20_ concentration (Figure 3B).

### 3.5. Repellent Effect of Essential Oils

**Figure 4:**
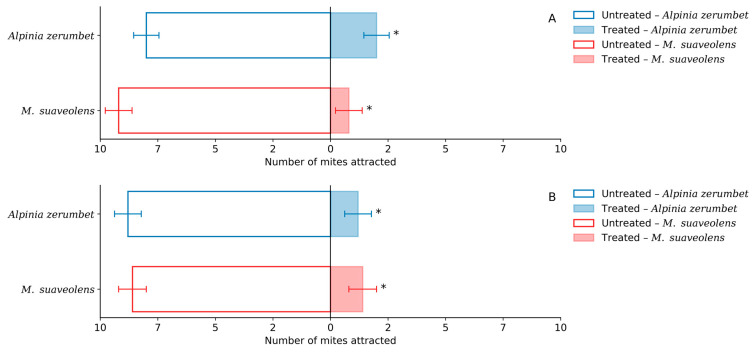
Number of *T. urticae* mites attracted to treated and untreated leaf disk areas after exposure to the LC_20_ (**A**) and LC_30_ (**B**) concentrations of essential oils from *A. zerumbet* and *M. suaveolens*. * Significant differences between treatments according to the Chi-square test (*χ^2^*, *p* < 0.05). n = 200; *p* < 0.0001.

Based on the analysis of mean repellency indices (RI), all evaluated concentrations yielded values below 1-DP, indicating a repellent classification. Accordingly, the essential oils of *A. zerumbet* and *M. suaveolens* at the sublethal concentrations of LC_20_ and LC_30_ were classified as repellents (Table 4). The analysis of the mean number of mites attracted to leaf disks treated with essential oils revealed that both oils exhibited significant repellent activity at LC_20_ and LC_30_ when compared to their respective untreated controls (Figure 4A,B).

At the LC_20_ concentration, *A. zerumbet* oil attracted an average of 2.0 mites, whereas the control attracted 8.0, representing a 75% reduction and a repellency percentage of 75%. *M. suaveolens* oil attracted an even lower number of mites (0.8) compared to 9.2 in the control, resulting in a repellency of approximately 91.3%.

A similar trend was observed at LC_30_. The mean number of mites attracted by *A. zerumbet* oil was 1.2, compared to 8.8 in the control, indicating a repellency of around 86.4%. *M. suaveolens* attracted 1.4 mites on average, versus 8.6 in the control, achieving a repellency percentage of 83.7%.

These results demonstrate that although both oils exhibited significant repellent activity against *T. urticae*, *M. suaveolens* was more effective at LC_20_ (Figure 4A), while *A. zerumbet* showed slightly greater repellency at LC_30_ (Figure 4B).

### 3.6. Multivariate Assessment of Lethal and Sublethal Effects on T. urticae: PCA of Experimental Parameters

**Figure 5:**
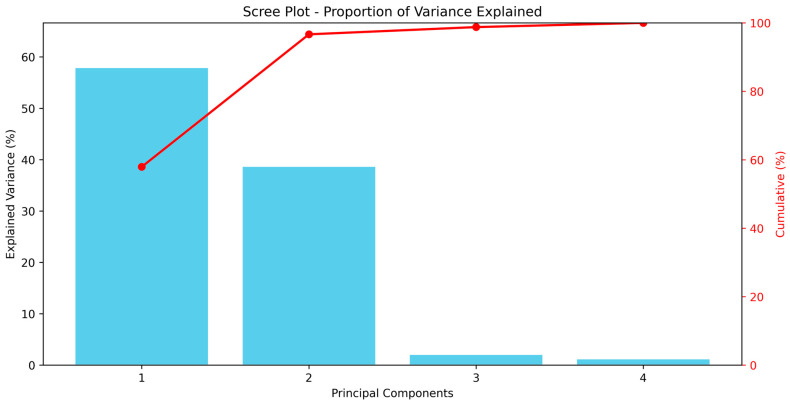
Scree plot showing the proportion of variance explained by each principal component in the PCA of lethal and sublethal effects on *T. urticae*.

The PCA results indicated that the first two principal components (PC1 and PC2) explained 96.65% of the total variance, with PC1 accounting for 57.94% and PC2 for 38.71%. Principal components PC3 and PC4 contributed only 2.11% and 1.24% of the variance, respectively, suggesting that the majority of the data variability was explained by the first two components (Figure 5).

**Figure 6:**
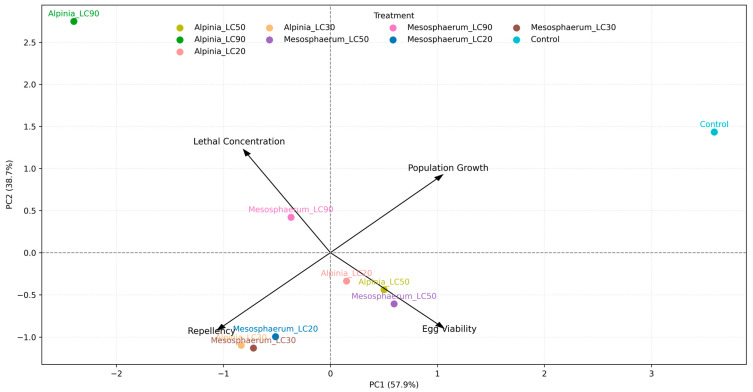
Biplot of the first two principal components (PC1 and PC2) showing the relationship between treatments and parameters related to lethal and sublethal effects on *T. urticae*.

The variables egg viability and population growth were more closely associated with PC2, whereas lethal concentration and repellency were predominantly associated with PC1 (Figure 6). This indicates that lethal concentration and repellency contributed more to the discrimination of treatments along the first principal component, which explained most of the overall variance. These results suggest that increased lethal concentrations also tend to enhance repellency, even though these parameters are related to distinct biological effects—lethal and sublethal, respectively.

**Figure 7:**
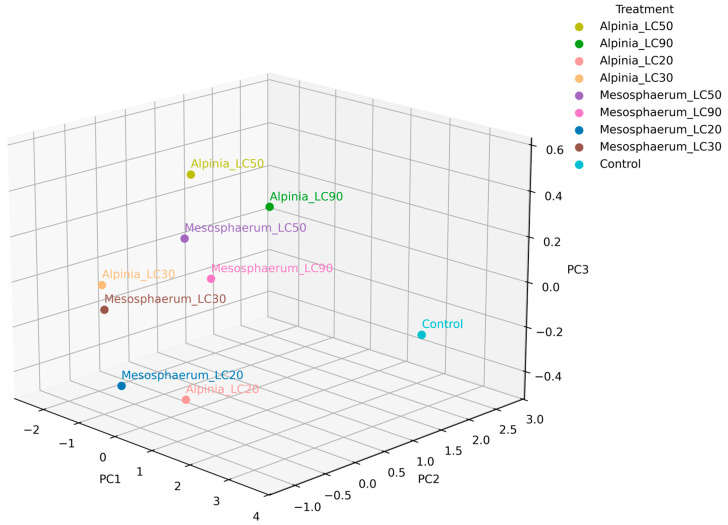
Three-dimensional PCA plot of principal components 1, 2, and 3 showing treatment differentiation based on lethal and sublethal effects on *T. urticae*.

The 3D comparison of the three principal components with the highest contributions to the total variance revealed that the lethal concentrations (LC_90_) of both essential oils were positioned farthest from the control, indicating a stronger effect on mite mortality (Figure 7). The treatments *Alpinia*_LC_90_ and *Mesosphaerum_*LC_90_, for instance, contributed most to PC1 and were clearly positively correlated with both lethal concentration and increased repellency, reinforcing their potential for mite management. Conversely, the LC_50_, LC_30_, and LC_20_ treatments were more associated with egg viability and population growth, highlighting the relevance of these concentrations beyond direct mortality effects.

## 4. Discussion

The essential oil of *A. zerumbet* showed considerable acaricidal activity against *T. urticae*, with LC_50_ and LC_90_ values of 8.74 µL/mL and 46.24 µL/mL, respectively, signifying substantial effectiveness in laboratory settings against adult female mites. Barbosa et al. [15] reported the chemical composition of this oil through GC-MS analysis, noting the presence of 1,8-cineol (16.13%) and sabinene (10.41%), values that are comparable to those observed in the current study, which recorded 1,8-cineole at 14.05% and sabinene at 12.6%. Jezler et al. [16] examined the essential oil derived from A. *zerumbet* leaves and determined 1,8-cineol as the second most abundant component, comprising 19.35% of the oil, subsequent to terpinen-4-ol. These findings validate the reliability of 1,8-cineole as a principal chemical component of the species, while its concentration may fluctuate based on botanical origin, plant organs investigated, and environmental factors. In the context of life-stage selectivity, the comparatively higher proportion of oxygenated monoterpenes in *A. zerumbet* (notably 1,8-cineole) provides a plausible basis for stronger ovicidal action, because small, volatile molecules are more likely to diffuse across egg envelopes and affect early embryogenesis in mites, as reported for 1,8-cineole–rich materials [17,18].

1,8-Cineol, or eucalyptol, is an oxygenated monoterpene renowned for its insecticidal and acaricidal properties. Hu et al. [19] found that this chemical induces significant pathogenic changes in mite cells, including nuclear membrane destruction, chromatin breakdown, and mitochondrial disruption. These effects result in the dysregulation of essential nervous system enzymes in mites, including acetylcholinesterase (AChE), monoamine oxidase (MAO), and nitric oxide synthase (NOS), alongside the induction of oxidative stress via heightened activity of antioxidant enzymes such as superoxide dismutase (SOD) and glutathione S-transferase (GSTs) [19]. The essential oil of *A. zerumbet* demonstrated significant toxicity towards adult female *T. urticae* in this study, with 1,8-cineol identified as a principal component, suggesting that analogous enzymatic and cytotoxic mechanisms may have facilitated the observed lethal effects. Consequently, the oil’s toxicity may be linked to disruption of essential cellular and enzymatic functions, especially given the mite’s nervous system’s susceptibility to oxygenated monoterpenes. Taken together with the ovicidal data, these mechanisms align with the notion that 1,8-cineole can act at multiple stages, with its volatility and small size favoring egg penetration while still contributing to adult toxicity [17,18,20].

Roh et al. [21] indicated that 1,8-cineole and limonene in essential oils from *Eucalyptus* species exhibited acaricidal and repellent properties against *T. urticae*, resulting in notable decreases in oviposition and heightened death in exposed females. Afify et al. [20] also showed that eucalyptus essential oils, abundant in 1,8-cineol, demonstrated harmful effects on both the eggs and adult females of *T. urticae*, associated with modifications in the activity of detoxifying enzymes, specifically glutathione S-transferase (GST) and esterases. The results support the idea that oxygenated monoterpenes, like 1,8-cineole, which make up 16.13% of *A. zerumbet* essential oil, could cause similar toxic effects in the mite. Considering the high death rate we observed and the oil’s chemical makeup, it seems likely that *T. urticae*’s exposure to the oil’s ingredients caused oxidative stress, which might have triggered or upset GST activity, making it harder for the mite to deal with the harmful effects of the volatile compounds. This disruption of the endogenous detoxification mechanism may have contributed to the reported toxicity, particularly during vulnerable life phases such as in adult females. By contrast, adult-selective toxicity can be strengthened by compounds that act predominantly via contact and membrane disruption with lower volatility, helping explain why *M. suaveolens*—richer in oxygenated sesquiterpenes—was more potent against adults.

Alongside 1,8-cineole, sabinene—a hydrocarbon monoterpene also found in *A. zerumbet* oil—demonstrates significant biological activity. Attia et al. [22] indicated that sabinene and other monoterpenes found in essential oils contributed to heightened mortality and reduced fertility in *T. urticae* when administered either singularly or in combinations. Moreover, the presence of oxygenated sesquiterpenes like β-eudesmol and caryophyllene oxide, as revealed in the chemical profile of *A. zerumbet* in this work, may augment the mechanisms of action against the mite. Such multi-constituent mixtures can yield stage-specific outcomes: monoterpenes (e.g., 1,8-cineole, sabinene) are more likely to influence egg viability and early development, whereas heavier sesquiterpenes (e.g., β-eudesmol) may contribute proportionally more to adult mortality.

The composition of the essential oil of *M. suaveolens* highlights a predominance of monoterpenes (e.g., β-sabinene, 1,8-cineol) and oxygenated sesquiterpenes (e.g., spathulenol, β-eudesmol), suggesting the potential for multifaceted biological activity. Notably, spathulenol—abundant in *M. suaveolens*—has documented acaricidal activity and, given its higher molecular weight and lower volatility relative to 1,8-cineole, may act preferentially on adults via contact/fumigant routes and enzyme/neural targets [23,24], which is consistent with our adult LC patterns.

When comparing the current findings to previous studies, Bezerra et al. [25] identified β-caryophyllene (18.57%), sabinene (15.94%), and spathulenol (11.09%) as the principal constituents of *M. suaveolens* oil, whereas Araújo Luz et al. [26] recognized 1,8-cineole as the major compound across all seasonal samples, with concentrations varying from 30.15% to 64.44%. Conversely, the concentration of 1,8-cineole recorded in this study (11.01%) was significantly lower, suggesting substantial variability affected by environmental and geographical factors. In this study, the spathulenol content (12.28%) surpassed that reported by Araújo Luz et al. [26], where it was detected solely during the rainy season at a significantly lower concentration (1.22%).

Despite β-sabinene being the predominant component in this study, there are currently no particular studies examining its acaricidal efficacy as a principal chemical against *T. urticae*. Consequently, emphasis should be placed on spathulenol, which has been more thoroughly defined in this context. Born et al. [23] assessed the acaricidal properties of isolated spathulenol and established its toxicity against *T. urticae* in both fumigation and contact experiments, affirming its significance as a principal bioactive compound. Perumalsamy et al. [24] identified spathulenol as a sesquiterpenoid with significant acaricidal properties, thereby reinforcing its importance in the oil’s bioactivity, especially against the home dust mite *Dermatophagoides farinae*.

Considering the significant toxicity identified in this study and the notable concentration of spathulenol in the oil, it is reasonable to deduce that this sesquiterpene plays a central role in the lethal effects against *T. urticae*. The mechanism may entail the disruption of neural or enzymatic pathways, potentially analogous to those characterized for other oxygenated sesquiterpenes. The simultaneous presence of additional minor sesquiterpenes, including elemol, viridiflorol, and β-eudesmol, may synergistically augment the overall acaricidal efficacy, thereby intensifying the oil’s toxicity through additive or potentiating interactions. In this framework, *M. suaveolens* chemotype (sesquiterpene-rich) would be expected to excel against adults, while *A. zerumbet* (with more 1,8-cineole) would be expected to excel against eggs—precisely the pattern observed here.

The mechanistic interpretations (e.g., enzymatic disruption and oxidative stress) are grounded in prior reports on 1,8-cineole and related terpenoids [19,20] and are presented here as hypotheses; no biochemical assays were performed in the present study. Follow-up work will quantify AChE, GST and esterase activities, oxidative-stress markers (SOD, CAT, lipid peroxidation), and—where feasible—transcriptional responses in eggs and adults exposed to each oil to directly test these mechanisms.

The ovicidal efficiency of *A. zerumbet* and *M. suaveolens* essential oils against *T. urticae* showed varying degrees of effectiveness. At the LC_90_ concentration, *A. zerumbet* oil markedly diminished egg viability to merely 2%, while *M. suaveolens* oil only permitted 57% viability, demonstrating a distinctly greater ovicidal impact for A. zerumbet under the evaluated conditions. At the LC_50_ level, both oils failed to produce a significant difference to the untreated control, indicating that elevated amounts are necessary to elicit considerable embryotoxic effects. Fatemikia et al. [17] evaluated the essential oil of *Elettaria cardamomum* (Zingiberaceae), a plant within the same botanical family as *A. zerumbet*, which contains 1,8-cineole as a principal component, and recorded an LC_50_ of 8.82 µL/L air against *T. urticae* eggs. Ayllón-Gutiérrez et al. [18] found that a nanoemulsion of 1,8-cineol greatly diminished the survival of *T. urticae* eggs, underscoring the ovicidal properties of this monoterpene.

Although *M. suaveolens* exhibited a lower ovicidal effect, the presence of 1,8-cineol (11.01%) in its composition may have contributed to the reduction in egg hatchability observed at the LC_90_ level. This compound likely played a supporting role in the observed bioactivity, although it was present at a slightly lower concentration than in *A. zerumbet* (14.05%). Furthermore, the presence of spathulenol (12.28%) may have enhanced the effect, despite the overall ovicidal performance being inferior to that of *A. zerumbet*.

*Alpinia zerumbet* had a stronger ovicidal effect due to its increased 1,8-cineol concentration and synergistic action with other bioactive compounds like sabinene (12.6%) and β-eudesmol (4.5%). Similar patterns have been seen in other species of the Zingiberaceae family, where complex combinations of monoterpenes and sesquiterpenes exhibit increased bioactivity. Reinforcing this, Li et al. [27] demonstrated that lemongrass (*Cymbopogon citratus*) oil, abundant in citral, markedly impeded the egg hatching of *Sarcoptes scabiei*, thereby supporting the general finding that oxygenated terpenoids might interfere with embryonic development in mites. Both essential oils exhibited ovicidal effects at elevated concentrations; however, *A. zerumbet* demonstrated significantly greater efficacy than *M. suaveolens*, likely attributable to its unique composition and equilibrium of active oxygenated terpenoids, notably 1,8-cineole, along with synergistic interactions among its components. The egg–adult divergence is therefore interpreted not as an inconsistency but as a chemotype-specific outcome, consistent with constituent modes of action reported in the literature [20,23,24,25,26,27].

The essential oils of *A. zerumbet* and *M. suaveolens* have shown inhibitory effects on the population growth of *T. urticae*, even at sublethal concentrations. No significant difference was noted between the two oils; nevertheless, both diminished the instantaneous rate of population expansion (ri) relative to the untreated control. The measured values of ri in this study varied from 0.4746 to 0.5155 in the treatments, compared to 0.5848 in the control, indicating a moderate inhibition of population growth due to essential oil exposure. Tsolakis and Ragusa [28] noted a significant reduction in ri, from 0.68 in the control group to merely 0.07 in mites subjected to a combination of caraway essential oil and potassium salts of fatty acids—this decline is markedly more pronounced than that observed in the current study, yet aligns with the premise that essential oils can impair reproductive metrics and postpone developmental processes.

Musa et al. [29] also reported that treatment with extracts of *Chenopodium ambrosioides* decreased the ri of *T. urticae* from 0.317 in the control group to 0.222 in the treated groups. Despite their overall lower values compared to the present study, the decrease trend is analogous, indicating that the essential oils of *A. zerumbet* and *M. suaveolens* similarly affect the reproductive capacity and longevity of the mites across successive generations. In a comparable study, Andrade et al. [30] demonstrated that *Varronia curassavica* essential oil significantly reduced *T. urticae* ri as concentration increased and also decreased oviposition and egg hatch at higher concentrations, underscoring significant sublethal effects. Although the decreases observed in the current study were not as significant, the pattern is evident: plant-derived chemicals, especially those abundant in monoterpenes and sesquiterpenes such as 1,8-cineol and spathulenol, might influence mite population growth.

Collectively, these findings underscore the potential of essential oils as both acute toxic agents and instruments for the long-term regulation of phytophagous mite populations. The consistent yet moderate reductions in ri observed suggest that *A. zerumbet* and *M. suaveolens* oils may be incorporated into pest management strategies aimed at diminishing the reproductive capacity and population growth of *T. urticae*, particularly when employed preventively or alongside other compatible control methods.

The repellency assays indicated that both *A. zerumbet* and *M. suaveolens* essential oils elicited significant behavioral avoidance in *T. urticae*, with repellency rates surpassing 75% at all evaluated sublethal concentrations. *M. suaveolens* had greater efficacy at LC_20_, but *A. zerumbet* showed marginally superior repellency at LC_30_, suggesting that the behavioral response may be dose-dependent and affected by variations in oil composition.

Several studies support the observed repellency and help interpret the potential roles of key constituents. Câmara et al. [31] evaluated the essential oil of *Croton rhamnifolioides* from different localities and estimated RC_50_/RC_90_ values for the oils against *T. urticae*. Repellency varied with chemotype, and the authors related these differences to changes in the relative abundance of constituents such as β-caryophyllene and 1,8-cineole. In our material, 1,8-cineole, β-caryophyllene, camphor, and spathulenol are present in differing proportions (with spathulenol reaching 12.28% in *M. suaveolens*), which likely explains the dose-dependent behavioral avoidance observed here—i.e., repellency arising from the combined action and relative proportions of constituents rather than from any single molecule alone.

Tak and Isman [32] also highlighted that terpenoid-rich essential oils, such as 1,8-cineol, sabinene, and α-terpineol, exhibit varied repellency based on their interactions within combinations. They noted that specific binary blends exhibited either enhanced or diminished repellent properties compared to individual chemicals, corroborating the concept that the efficacy demonstrated in our investigation may stem from synergistic interactions among constituents. Sousa et al. [33] further emphasized the dual role of essential oils in providing both lethal and repellent effects against phytophagous mites, reinforcing their relevance in sustainable pest management strategies.

Zhang et al. [34] discovered that *Rosmarinus officinalis* oil, which contains 25.2% 1,8-cineol, attained a repellency rate of 92.67% against *Aleuroglyphus ovatus*, hence reinforcing the significant behavioral impact of this monoterpene. Fatemikia et al. [17] similarly observed that *E. cardamomum* oil, which is also abundant in 1,8-cineole, achieved a 67.9% repellency against *T. urticae*, albeit at higher doses than those examined in this study.

The elevated repellency levels observed in this investigation indicate that both *A. zerumbet* and *M. suaveolens* oils disrupt host location behavior in *T. urticae*. The enhanced performance of *M. suaveolens* at LC_20_ may be attributed to its distinct chemical composition, particularly the synergy between 1,8-cineol and spathulenol. These findings underscore the efficacy of these oils as natural repellents in integrated pest management strategies, especially for the behavioral disruption of phytophagous mites. Because essential oils are volatile and susceptible to photodegradation, semi-field and field validations are planned. Greenhouse trials will evaluate persistence of adulticidal, ovicidal and repellent effects on plants at 0, 1, 3, 5 and 7 days after application, compare conventional sprays with nanoemulsified/encapsulated formulations, and assess phytotoxicity and compatibility with predatory mites; complementary GC–MS of leaf washings will track residue weathering. These data will inform dose, interval and formulation requirements for IPM deployment.

The multivariate analysis offered a comprehensive perspective on the deadly and sublethal impacts of essential oils in *T. urticae*, identifying the biological characteristics that most significantly influenced the differential across treatments. The data distribution in the multivariate space emphasized the significance of both direct effects, including mortality and repellency, and sublethal influences on reproduction and population growth dynamics.

Higher concentrations showed greater efficacy in eliciting immediate effects on the pest, however lower concentrations were notable for their physiological and behavioral implications that undermine population dynamics over time. The findings validate that the integration of several effects—lethal and sublethal—constitutes a viable and sustainable approach for managing *T. urticae*, hence enhancing the relevance of the evaluated essential oils in integrated pest management initiatives.

Evaluating the cumulative effects of toxicity, ovicidal properties, repellency, and impacts on population dynamics, the essential oils of *A. zerumbet* and *M. suaveolens* present themselves as viable botanical options for the control of *T. urticae*. The facts endorse their application at elevated concentrations, specifically LC_90_, for the prompt diminution of mite populations via direct mortality and ovicidal effect. The use of sublethal concentrations (LC_20_–LC_30_) may concurrently facilitate long-term suppression, limiting population growth and causing repellency.

## 5. Conclusions

Essential oils of *Alpinia zerumbet* and *Mesosphaerum suaveolens* showed multitarget acaricidal activity against *Tetranychus urticae*: *M. suaveolens* was more potent on adults, whereas *A. zerumbet* was stronger on eggs. At sublethal doses, both oils repelled mites and suppressed population growth; PCA indicated that lethal concentration and repellency were the main drivers of efficacy. These properties support their use as botanical tools within IPM and motivate further work on formulation and chemotype standardization.

## Figures and Tables

**Table 1:** Chemical characterization of the essential oil of *A. zerumbet*.

Compound	Retention Time	LRI Obtained	LRI Literature	Identification Method ^1^	%	CAS
**α-thujene**	2.34	889	926	NIST + LRI	1.68	2867-05-02
**α-pinene**	2.38	896	933	NIST + LRI	1.52	127-91-3
**sabinene**	2.60	940	973	NIST + LRI	12.62	3387-41-5
**β-cymene**	2.86	995	1023	NIST + LRI	6.87	535-77-3
**1,8-cineol**	2.90	1002	1032	NIST + LRI	14.05	470-82-6
**vinyl hexanoate**	3.10	1043	-	NIST ^2^	2.96	3050-69-9
**p-menth-8-en-1-ol**	3.24	1070	1147	NIST + LRI	1.65	7299-41-4
**terpin-3-en-1-ol**	3.43	1110	1120	NIST + LRI	1.32	586-82-3
**bicyclo [3.1.0]hexan-2-one**	3.51	1126	1147	NIST + LRI	1.4	513-20-2
**terpinen-4-ol**	3.60	1144	1179	NIST + LRI	6.03	562-74-3
**ascaridole**	3.80	1185	1257	NIST + LRI	1.87	512-85-6
**elemol**	5.05	1457	1542	NIST + LRI	0.83	639-99-6
**nerolidol**	5.08	1465	1535	NIST + LRI	0.74	142-50-7
**caryophyllene oxide**	5.19	1498	1582	NIST + LRI	2.71	1139-30-6
**γ-eudesmol**	5.35	1545	1630	NIST + LRI	1.1	1209-71-9
**β-eudesmol**	5.42	1567	1649	NIST + LRI	4.5	473-15-4

^1^ NIST = Identification performed using the NIST mass spectrometry library; LRI = identification based on linear retention index. ^2^ Spectral similarity equal to 97.

**Table 2:** Chemical characterization of the essential oil of *M. suaveolens*.

Compound	Retention Time	LRI Obtained	LRI Literature	Identification Method ^1^	%	CAS
**α-pinene**	2.38	896	933	NIST + LRI	1.91	80-56-8
**β-sabinene**	2.60	941	977	NIST + LRI	15.78	3387-41-5
**1,8-cineol**	2.90	1002	1039	NIST + LRI	11.01	470-82-6
**fenchone**	3.18	1060	1086	NIST + LRI	2.27	126-21-6
**fenchol**	3.31	1085	1110	NIST + LRI	1.93	1632-73-1
**terpinen-4-ol**	3.60	1145	1173	NIST + LRI	0.96	562-74-3
**β-bourbonene**	4.46	1321	1384	NIST + LRI	11.13	5208-59-3
**4,11,11-trimethyl-8-methylene-bicyclo [7.2.0]undec-3-ene**	4.60	1348	1460	NIST + LRI	0.32	889360-49-0
**elemol**	5.05	1457	1542	NIST + LRI	1.22	639-99-6
**spathulenol**	5.17	1492	1548	NIST + LRI	12.28	6750-60-3
**viridiflorol**	5.22	1508	1589	NIST + LRI	0.63	552-02-3
**β-eudesmol**	5.42	1568	1649	NIST + LRI	8.52	473-15-4

^1^ NIST = Identification performed using the NIST mass spectrometry library; LRI = identification based on linear retention index.

**Table 3:** Toxicity effect of *A. zerumbet* and *M. suaveolens* essential oils on *T. urticae*.

			Slope (±SE)	LC_50_		LC_90_			
Essential Oil	n	DF		(CI95%)	TR_50_	(IC95%)	TR_90_	χ^2^	*p*
*M. suaveolens*	750	3	2.32 ± 0.21	4.24	2.06	15.08	3.06	3.36	0.33
				(3.41–5.25)		(11.50–21.49)			
*A. zerumbet*	750	3	1.77 ± 0.26	8.74	-	46.24	-	5.91	0.11
				(6.82–10.85)		(31.38–90.88)			

n = number of insects used in the test; DF = degrees of freedom; SE = standard error of the mean; CI = confidence interval; TR = toxicity ratio; χ^2^ = chi-square; *p* = probability.

**Table 4:** Repellent activity of *A. zerumbet* and *M. suaveolens* essential oils against *T. urticae*.

Essential Oil	Lethal Concentration	RI (M ± SD)	Classification
*M. suaveolens*	LC_20_	(0.16 ± 0.14)	Repellent
	LC_30_	(0.28 ± 0.27	Repellent
*A. zerumbet*	LC_20_	(0.40 ± 0.24)	Repellent
	LC_30_	(0.24 ± 0.22)	Repellent

RI (Repellency Index) = 2G/(G + P), where G = number of mites attracted to the treatment; P = number of mites attracted to the control; M = mean; SD = standard deviation.

## Data Availability

The original contributions presented in this study are included in the article. Further inquiries can be directed to the corresponding author.
